# The Influence of Social Distancing on COVID-19 Mortality in US Counties: Cross-sectional Study

**DOI:** 10.2196/21606

**Published:** 2021-03-18

**Authors:** Phoebe Tran, Lam Tran, Liem Tran

**Affiliations:** 1 Chronic Disease Epidemiology Department Yale School of Public Health Yale University New Haven, CT United States; 2 Department of Biostatistics Michigan School of Public Health University of Michigan Ann Arbor, MI United States; 3 Department of Geography University of Tennessee Knoxville, TN United States

**Keywords:** COVID-19, marginal effects, mortality, negative binomial model, social distancing

## Abstract

**Background:**

Previous studies on the impact of social distancing on COVID-19 mortality in the United States have predominantly examined this relationship at the national level and have not separated COVID-19 deaths in nursing homes from total COVID-19 deaths. This approach may obscure differences in social distancing behaviors by county in addition to the actual effectiveness of social distancing in preventing COVID-19 deaths.

**Objective:**

This study aimed to determine the influence of county-level social distancing behavior on COVID-19 mortality (deaths per 100,000 people) across US counties over the period of the implementation of stay-at-home orders in most US states (March-May 2020).

**Methods:**

Using social distancing data from tracked mobile phones in all US counties, we estimated the relationship between social distancing (average proportion of mobile phone usage outside of home between March and May 2020) and COVID-19 mortality (when the state in which the county is located reported its first confirmed case of COVID-19 and up to May 31, 2020) with a mixed-effects negative binomial model while distinguishing COVID-19 deaths in nursing homes from total COVID-19 deaths and accounting for social distancing– and COVID-19–related factors (including the period between the report of the first confirmed case of COVID-19 and May 31, 2020; population density; social vulnerability; and hospital resource availability). Results from the mixed-effects negative binomial model were then used to generate marginal effects at the mean, which helped separate the influence of social distancing on COVID-19 deaths from other covariates while calculating COVID-19 deaths per 100,000 people.

**Results:**

We observed that a 1% increase in average mobile phone usage outside of home between March and May 2020 led to a significant increase in COVID-19 mortality by a factor of 1.18 (*P*<.001), while every 1% increase in the average proportion of mobile phone usage outside of home in February 2020 was found to significantly decrease COVID-19 mortality by a factor of 0.90 (*P*<.001).

**Conclusions:**

As stay-at-home orders have been lifted in many US states, continued adherence to other social distancing measures, such as avoiding large gatherings and maintaining physical distance in public, are key to preventing additional COVID-19 deaths in counties across the country.

## Introduction

With the rapid spread of COVID-19 across the United States in early 2020, many states began enacting social distancing measures (stay-at-home orders, maintaining physical distance in public, and avoiding large gatherings) from March 2020 [1]. Enforcement of social distancing measures varied widely throughout the United States with some states including Arkansas, Iowa, Nebraska, North Dakota, Oklahoma, South Dakota, Utah, and Wyoming opting to not implement stay-at-home orders altogether [1]. Even with social distancing measures in place in most parts of the country, over 108,000 COVID-19 deaths were reported by the end of May 2020 [2]. As stay-at-home orders have been lifted in most US states and numbers of COVID-19 cases continue to rise, it is critical to assess the role of social distancing in preventing COVID-19 deaths in the United States [1].

Although some studies have assessed the influence of social distancing on COVID-19 mortality in the United States with actual rather than simulated data, these studies—including those performed by Medline et al [3] and Siedner et al [4]—have examined this relationship at the national level but not at a more granular geographic scale. Furthermore, these 2 studies [3,4] do not consider mortality directly but rather consider tangentially related mortality measures (change in the mortality growth rate and the period between the report of the first confirmed COVID-19 case and the peak number of deaths), and the study by Medline et al [3] does not include all US states. Additionally, Medline et al [3] did not separate COVID-19 deaths in nursing homes—a major (~30%) source of all COVID-19 fatalities—from total COVID-19 deaths, which could have potentially led to biased findings that are not representative of the ongoing pandemic [3,4]. These approaches may obscure the actual effectiveness of social distancing and differences in social distancing behaviors by county.

Consequently, we sought to bridge the gap in studies on COVID-19–related mortality in the United States by investigating the association between social distancing and COVID-19 mortality at the county level across the United States while simultaneously separating COVID-19 deaths in nursing homes from total COVID-19 deaths and accounting for social distancing and COVID-19–related factors. To accomplish this, we modeled the relationship between the average proportion of mobile phone usage outside of home during the time when stay-at-home orders were in place nationwide (a proxy variable for social distancing) and COVID-19 mortality by using a mixed-effects negative binomial model and marginal effects at the mean.

## Methods

### Assessment of COVID-19 Mortality

Data on deidentified confirmed COVID-19 deaths in all 3142 US counties were obtained from the Coronavirus Resource Center of the Center for Systems Science and Engineering at Johns Hopkins University [5]. This data set contains comprehensive information on COVID-19 death counts provided by the US Centers for Disease Control and Prevention (CDC) and state health departments [5]. Additionally, we retrieved deidentified data on COVID-19 deaths in US nursing homes from the COVID-19 nursing home data of the Centers for Medicare and Medicaid Services [6]. We then used ArcGIS Pro 2.5 [7] to identify all COVID-19 deaths in nursing homes at facilities within a county’s geographic boundaries for all US counties. We then defined a county’s COVID-19 mortality (deaths per 100,000 people) from the time a county reported its first confirmed case of COVID-19 and up to May 31, 2020, as follows:

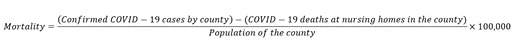



We selected May 31, 2020, as the study endpoint as most social distancing orders by state health departments in the United States were enforced in late March to early April until mid- and late May of 2020 [1]. COVID-19 deaths in nursing homes were excluded from a county’s total number of confirmed COVID-19 deaths because social distancing measured through mobile phone tracking is not an accurate measure of social distancing behavior among nursing home residents [8].

### Social Distancing Metrics

County-level social distancing metrics used in this study were derived from the SafeGraph COVID-19 Consortium [8], which provides social distancing data curated through anonymous global positioning system–based tracking of mobile phones [8]. We used the average proportion of mobile phone usage outside of home between March and May 2020 as our social distancing measure as this timespan encompasses the period of the enforcement of stay-at-home orders in most parts of the United States [1,8]. The average proportion of mobile phone usage outside of home in February 2020 was used to establish a baseline on which to compare the social distancing measure with that since February 2020 when the number of COVID-19 cases began to rapidly rise in the United States [9].

### Covariates

We included covariates that have been reported to be associated with social distancing and COVID-19 from previous studies in these areas [10-13]. These covariates were the number of days between the report of the first confirmed case of COVID-19 and May 31, 2020 (determined for each US county from Johns Hopkins University’s COVID-19 data set [5]); population density; social vulnerability; and hospital resource availability. With data on population and county size from the United States Census Bureau, we calculated a county’s population density by dividing the number of individuals in a county by that county’s area in square miles [14]. For population density, we used “100 persons per square mile” as the unit because the US population density at the county level varies across 5 orders of magnitude (eg, mean=270, median=40, minimum<1, and maximum>72,000), and multiples of hundreds of the average population density allow for easier interpretation of the results [15]. Social vulnerability was assessed using 15 measures that constitute the CDC’s social vulnerability index: proportion of the population below poverty line, unemployment rate, per-capita income, proportion of the population >25 years of age with no high school diploma, proportion of the population >65 years of age, proportion of the population <17 years of age, proportion of the civilian noninstitutionalized population with a disability, proportion of the population that is a single-parent household with children <18 years of age, proportion of the population that is a minority, proportion of the population >5 years of age who speak English “less than well,” proportion of housing that is a structure with >10 units, proportion of housing that is a mobile home, proportion of occupied housing units with more people than rooms, proportion of households with no vehicles, and the proportion of the population in institutionalized group quarters [16]. Data on hospital resource availability were obtained from the Environmental Systems Research Institute’s COVID-19 Resource Center [17]. Specifically, we used the number of staffed hospital beds within a county as our measure of hospital resource availability because this variable had complete data for the largest number of counties, being highly correlated with other measures of hospital resource availability such as the number of adult beds in the intensive care unit, number of licensed beds, and average ventilator use [17,18].

### Statistical Modeling

In order to assess the influence of social distancing on COVID-19 mortality, we developed a mixed-effects negative binomial model. Our choice of a mixed-effects negative binomial model was motivated by 2 factors observed during the data processing phase of our study: (1) considerable variation in the average state-level mortality rates, suggesting the necessity of a state-level random intercept and (2) the variance in county-level mortality rates in each state tending to be markedly greater than the average state-level mortality rate, suggesting the use of a negative binomial model. A county’s COVID-19 mortality up to May 31, 2020, was specified as the model outcome, while the social distancing exposure in the model was average proportion of mobile phone usage outside of home between March and May 2020. The aforementioned covariates associated with social distancing and COVID-19 were also included in the model for adjustment purposes. As larger counties are more likely to have a greater COVID-19 mortality than smaller counties, owing to their population size, the model included a population size offset to account for this tendency. Potential correlations resulting from counties within the same state and with comparable behavioral factors, health care systems, and COVID-19 testing policies were dealt with by including a random intercept by state in the model. Results from the negative binomial model are reported as incidence rate ratios, which were calculated by exponentiating the model’s coefficients. Using the same mixed-effects negative binomial model, we also performed an analysis that included COVID-19 deaths in nursing homes, and the results displayed considerable differences between the model that included COVID-19 deaths in nursing homes and the main model that excluded these deaths (Multimedia Appendix 1).

Based on the results of the mixed-effects negative binomial model, we examined the impact of social distancing on a county’s COVID-19 mortality apart from the influence of other factors through marginal effects at the mean. The marginal effects at the mean set all covariates besides the social distancing variable (average proportion of mobile phone usage outside of home between March and May 2020) to their average value [19]. Compared to traditional regression models, marginal effects at the mean facilitated clearer isolation from other factors involved in the effect of social distancing on COVID-19 deaths within a county [19]. Marginal effects analyses were performed for values of the average proportion of mobile phone usage outside of home of 25%-41% between March and May 2020 because this range contains the mean (31.73%) of the social distancing variable as well as both extremes of social distancing behavior [8]. All statistical analyses were carried out using Stata (version 15, StataCorp), and results were considered significant when Cronbach *α*=.05 [20].

### Data Availability

The COVID-19 and social distancing data used in this study are publicly available and can be found on the Center for Systems Science and Engineering at Johns Hopkins University’s website [5] and SafeGraph’s website [8].

## Results

On including COVID-19 deaths in nursing homes, the total number of COVID-19 deaths during the study period was 102,958, while the mean county-level COVID-19 mortality was 13 deaths per 100,000 people. Modeling results for the social distancing variable and other covariates of interest are presented in Table 1. With respect to our social distancing variable, for every 1% increase in the average proportion of mobile phone usage outside of home between March and May 2020, COVID-19 mortality—in terms of deaths per 100,000 people—was expected to significantly increase by a factor of 1.18 (*P*<.001). This is in contrast with the variable in February, where for every 1% increase in the average proportion of mobile phone usage outside of home in February, COVID-19 mortality significantly decreased by a factor of 0.90 (*P*<.001). Coefficient estimates for population density and the number of days between the report of the first confirmed case of COVID-19 and May 31, 2020, were also significant. For every extra 100 individuals per square mile, the expected COVID-19 mortality was projected to increase by a factor of 1.02 (1.0002^100^), whereas for every extra day between the first confirmed case of COVID-19 and May 31, 2020, the expected COVID-19 mortality increased by a factor of 1.03.

**Table 1:** Mixed-effects negative binomial model examining the impact of social distancing on COVID-19 mortality (deaths per 100,000 people) in US counties between the period of the report of the first case of COVID-19 and May 31, 2020.

Variables	Incidence rate ratio (95% CI)	*P* value
Average proportion of mobile phone usage outside of home between March and May 2020	1.18 (1.12-1.24)	<.001
Average proportion of mobile phone usage outside of home in February 2020	0.90 (0.86-0.94)	<.001
Population density (100 persons per square mile)	1.02 (1.01-1.04)	<.001
Days between the report of the first confirmed case of COVID-19 and May 31, 2020	1.03 (1.02-1.04)	<.001

**Figure 1:**
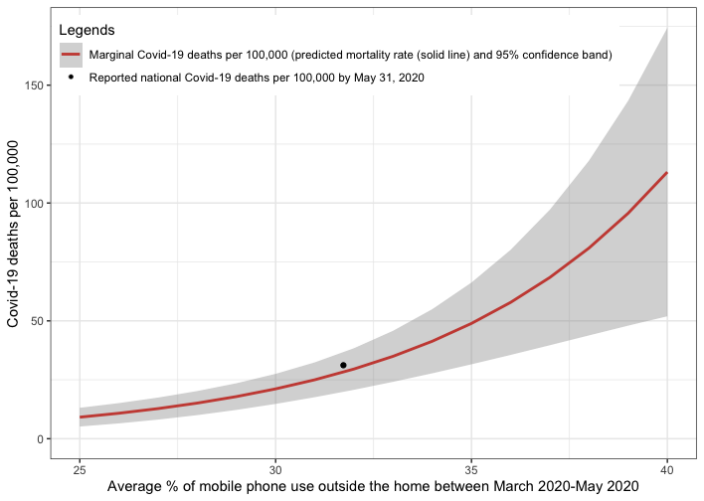
Predicted COVID-19 deaths per 100,000 people in the United States vs the average proportion of mobile phone usage outside of the nursing home between March and May 2020.

We plotted the expected nationwide COVID-19 mortality rates in Figure 1 for a range of proportions of mobile phone usage outside of home, corresponding to different stringencies of lockdown measures and adherence to social distancing. The red line represents the marginal predicted nationwide deaths per 100,000 people, with the shaded band indicating the 95% CI around it. The black dot represents the reported nationwide COVID-19 mortality rate by May 31, 2020, and falls well within the predicted interval. 

## Discussion

Our study examined the influence of social distancing on COVID-19 mortality across US counties while simultaneously accounting for COVID-19 deaths in nursing homes and covariates associated with social distancing and COVID-19. Social distancing, as assessed from the average proportion of mobile phone usage outside of home between March and May 2020 but not in February 2020, was found to correspond with a significant increase in COVID-19 mortality in a county. In addition, by estimating nationwide COVID-19 mortality rates by May 31, 2020, for a range of proportions of mobile phone usage outside of home, we found that COVID-19 mortality by this date could have been even higher had there been poorer adherence to social distancing.

While our finding of increased mobile phone usage outside of home during the period of enforcement of stay-at-home orders in many parts of the United States being associated with higher COVID-19 mortality is somewhat expected, it is of some interest that a significant negative association between the average proportion of mobile phone use outside of home in February 2020 and COVID-19 mortality was observed. In fact, high proportions of mobile phone usage outside of home were recorded in February 2020 more often in suburban and rural counties. As the first appearance of COVID-19 tended to be later in these less densely populated regions, COVID-19 mortality in February 2020 in suburban and rural counties was lower than that in urban regions where the virus had time to spread, which resulted in the negative association observed in February 2020.

Given the current prevalence and numerous ramifications of COVID-19, a few studies carried out in the United States [3,4] have examined the impact of social distancing on COVID-19 at the national level. Concurrent with Medline et al [3] and Siedner et al [4], we also determined that social distancing was a critical factor in reducing COVID-19 mortality. However, it is difficult to directly compare our findings with those of Medline et al [3] and Siedner et al [4] because the method of assessing social distancing and COVID-19 mortality varies between these 2 studies and our study. For instance, Medline et al [3] assessed social distancing on the basis of the period between the report of the first COVID-19 case and the implementation of social distancing measures and estimated COVID-19 mortality on the basis of the period between the report of the first COVID-19 case and the peak of COVID-19 deaths. They reported a positive association between social distancing and COVID-19 mortality where each additional day in implementing a stay-at-home order additionally contributed to the peak of COVID-19 deaths [3]. Siedner et al [4] assessed social distancing by comparing the number of COVID-19 cases 14 days before and 3 days after a stay-at-home order was implemented, and they considered the change in the COVID-19 mortality growth rate as the outcome. Thus, Siedner et al [4] reported that 1 week after stay-at-home orders were implemented, the COVID-19 mortality growth rate significantly decreased day by day by 2%. By including all US counties and separating COVID-19 deaths in nursing homes from total COVID-19 deaths, our results complement those of previous studies on social distancing and COVID-19 mortality in the United States while simultaneously providing additional information regarding this relationship at a more granular level.

Although our study provides novel estimates of the impact of social distancing on COVID-19 deaths, it still has limitations that need to be considered. Our study used a proxy variable for social distancing (average proportion of mobile phone usage outside of home during March-May 2020), which would be unable to track social distancing when individuals leave their phones at home or turn off their location services, and may not encompass some aspects of social distancing such as maintaining physical distance in public spaces and avoiding large group gatherings [8]. Although our metric for social distancing is incomplete, it is arguably one of the more quantitative approaches to track social distancing that may be prone to less bias relative to other metrics such as the dates of the implementation of stay-at-home orders or attendance at cultural, sporting, or religious events [2,21]. Additionally, approximately 95% of the adult population in the United States owns a mobile phone, with no significant deviations in ownership by race, age, education, income, or urban or rural residence [22]. Consequently, there is no evidence that any measurement error would systematically bias the social distancing coefficient either upwards or downwards and any random measurement error that does exist would not lead to any significant changes in the estimated covariate values. Finally, as with many studies on COVID-19 mortality in the United States, our study is limited by the quality of currently available data on COVID-19 mortality owing to a combination of factors including insufficient testing kits to properly diagnose COVID-19, suspected COVID-19 deaths that were instead attributed to other respiratory illnesses such as pneumonia and influenza, and delays in COVID-19 deaths being reported to the CDC [23,24].

The impact of social distancing on the COVID-19 pandemic has been particularly challenging to model at a national level. Different jurisdictions have imposed inconsistent policies with varying levels of restrictions, and substantial heterogeneity exists in the actual adherence to these social distancing guidelines. Furthermore, there is considerable variability in the availability and quality of COVID-19 mortality data. In this study, we considered daily county-level mobile phone usage outside of home and aggregated it into a national level data set to quantify the influence of social distancing on COVID-19 mortality. In doing so, we managed to account for within-county heterogeneity in the implementation of and adherence to social distancing measures. By including both mobile data usage outside of home from February 2020 (before social distancing measures) and during March-May 2020 (during and after social distancing measures), our study examined the entire span of stay-at-home orders in US states and provides a comprehensive overview of the role of social distancing in reducing COVID-19 deaths. Future studies are required to further examine the relationship between social distancing and COVID-19 given the relaxation of social distancing measures and the potential burden of continued COVID-19 mortality.

## Appendix

Multimedia Appendix 1 Results from the mixed-effects negative binomial model with nursing home COVID-19 deaths included.

## Abbreviations

CDC: Centers for Disease Control and Prevention
