# Supplementary material for: The Influence of Social Distancing on COVID-19 Mortality in US Counties: Cross-sectional Study
Source: JMIR Public Health Surveill. 2021 Mar 18;7(3):e21606. doi: 10.2196/21606 (PMC7977611; doi:10.2196/21606)
Supplement: Multimedia Appendix 1 [file publichealth_v7i3e21606_app1.docx]

**Appendix**

Results from the mixed-effects negative binomial model with nursing home Covid-19 deaths included.

| **Variables** | **Incidence Rate Ratio** | **95% Confidence Interval** | ***P*** |
| --- | --- | --- | --- |
| Average % of mobile phones leaving home between March 2020-May 2020 | 0.90 | (0.86, 0.95) | <.001 |
| Average % of mobile phones leaving home in February 2020 | 1.18 | (1.12, 1.24) | <.001 |
| Population density (100 persons per square mile) | 1.02 | (0.99, 1.03) | 0.091 |
| Days between when the first confirmed case was reported and May 31^st^, 2020 | 1.03 | (1.03, 1.04) | <.001 |
